# Zoonotic human liver flukes, a type 1 biocarcinogen, in freshwater fishes: genetic analysis and confirmation of molluscan vectors and reservoir hosts in Bangladesh

**DOI:** 10.1186/s40249-024-01209-0

**Published:** 2024-06-01

**Authors:** Sharmin Shahid Labony, Md. Abdul Alim, Muhammad Mehedi Hasan, Md. Shahadat Hossain, Sharmin Akter, Joydeep Paul, Thahsin Farjana, Md. Haydar Ali, Mohammad Zahangir Alam, Takeshi Hatta, Hayato Kawada, Keiko Mizutani, Naotoshi Tsuji, Anisuzzaman

**Affiliations:** 1 Department of Parasitology, Faculty of Veterinary Science, Bangladesh Agricultural University, Mymensingh, 2202 Bangladesh; 2 Department of Fisheries Technology, Faculty of Fisheries, Bangladesh Agricultural University, Mymensingh, 2202 Bangladesh; 3 Department of Biotechnology, School of Life Sciences & Biotechnology, Adamas University, Barasat - Barrackpore Rd, Jagannathpur, Kolkata, West Bengal 700126 India; 4 Department of Pathology and Parasitology, Hajee Mohammad Danesh Science and Technology University, Dinajpur, 5200 Bangladesh; 5 Department of Parasitology and Tropical Medicine, Kitasato University School of Medicine, 1- 15-1 Kitasato, Minami, Sagamihara, Kanagawa 252-0374 Japan; 6 Department of Molecular and Cellular Parasitology, Kitasato University Graduate School of Medical Sciences, 1-15-1 Kitasato, Minami, Sagamihara, Kanagawa 252-0373 Japan

**Keywords:** Human liver fluke, Freshwater fish, Molluscan vector, Reservoir host, Bangladesh

## Abstract

**Background:**

Opisthorchiid flukes, particularly *Opisthorchis viverrini*, *Opisthorchis felineus*, *Clonorchis sinensis*, and *Metorchis* spp. are the most common fish-borne zoonotic human liver flukes (hLFs). Liver fluke infections are more prevalent in resource-deprived and underprivileged areas. We herein estimated the prevalence of the metacercariae (MC) of major hLFs in common large freshwater fishes (lFWF) marketed for human consumption from some selected areas of Bangladesh along with detection of their molluscan vectors and reservoirs.

**Methods:**

The current status of fish-borne zoonotic hLF infections in lFWF was investigated along with their molluscan vectors and mammalian reservoir hosts in Mymensingh and Kishoreganj in Bangladesh from July 2018–June 2022 using conventional and multiple molecular techniques, such as PCR, PCR-restriction fragment length polymorphism (RFLP), sequencing, and bioinformatic analyses. The infection rate of fishes was analyzed using the *Z*-test and the loads of MC were compared using the chi-squared (*χ*^2^) test.

**Results:**

The MC of *C. sinensis*, *Opisthorchis* spp., and *Metorchis* spp. were detected in 11 species of common and popular lFWF. In lFWF, the estimated prevalence was 18.7% and the mean load was 137.4 ± 149.8 MC per 100 g of fish. The prevalence was the highest (*P* < 0.05) in spotted snakehead fishes (*Channa punctata*, 63.6%). The highest rate of infection (*P* < 0.05) was observed with the MC of *C. sinensis* (11.8%). Metacercariae were almost equally (*P* > 0.05) distributed between the head and body of fishes. The infection rate was slightly higher in cultured (19.6%) fishes. The MC of *C. sinensis*, *O. felineus*, *O. viverrini*, and *Metorchis orientalis* in fishes were confirmed using PCR, PCR-RFLP and bioinformatics. The cercariae of opisthorchiid (*Pleurolophocercus* cercariae) flukes were only recovered from *Bithynia* spp. (3.9%, 42 out of 1089). The ova of hLFs from dogs (4.3%, 5 out of 116) and cats (6.0%, 6 out of 100), and adult flukes (*M*. *orientalis*) from ducks (41.1% 113 out of 275) were detected.

**Conclusions:**

The MC of hLFs are highly prevalent in fresh water fishes in Bangladesh. Reservoir hosts, such as street dogs, cats, and ducks carried the patent infection, and residents of Bangladesh are at risk.

**Graphical Abstract:**

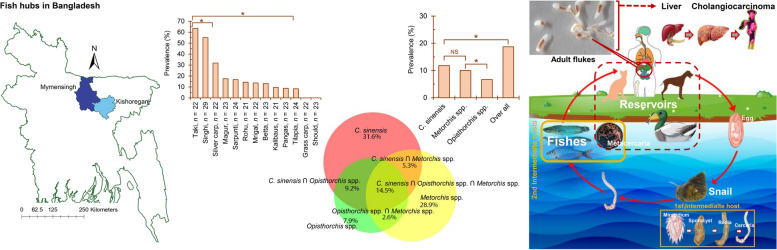

**Supplementary Information:**

The online version contains supplementary material available at 10.1186/s40249-024-01209-0.

## Highlights

■ Human liver flukes are fish-borne zoonotic trematodes prevalent in poor population.

■ Eleven species of common fresh water fishes were infected with metacercariae.

■ *Clonorchis sinensis*, *Opisthorchis felineus*, *O. viverrini* and *Metorchis orientalis* were confirmed.

■ Pleurolophocercus cercariae were recovered from *Bithynia* spp.

■ Ova of human liver flukes from dogs, and cats, and adult flukes from ducks were detected.

**Supplementary Information:**

The online version contains supplementary material available at 10.1186/s40249-024-01209-0.

## Background

Human liver fluke (hLF) infections caused by opisthorchiid flukes, such as *Opisthorchis viverrini*, *Opisthorchis felineus*, *Clonorchis sinensis*, and *Metorchis* spp., are neglected tropical diseases (NTD). Liver fluke infections are more prevalent in resource-deprived and underprivileged areas, particularly among the poorest segment of the global population [1]. These trematodal diseases are mainly transmitted by fishes and fish products and, thus, are known as fish-borne zoonotic trematodiasis (FZTs) [2]. These flukes have a complex life-cycle that requires three different species of hosts. A series of developmental stages occurs in gastropod snails, the first intermediate host. Cercariae emerge from snails and enter the freshwater fishes of different species, which act as the second intermediate host. Cercariae encyst into the infective stage (metacercaria, MC) in fishes, and humans along with other omnivorous or carnivorous mammals (e.g., cats and street dogs) act as definitive hosts, in which adult worms develop [1, 3]. Individuals who consume raw, poorly cooked, or salt-preserved fish containing viable MC become infected with these flukes [1, 2]. The consumption of food contaminated with MC through tableware may also result in infection [4]. Ingested MC excyst in the duodenum, and the juvenile worm migrates to the biliary tract through the ampulla of Vater [5], mature within 16–25 days, and live there for up to 30 years [6, 7]. Liver fluke infections are widespread throughout the world, particularly Southeast Asian countries [1]. Bangladesh is a highly populated, resource-constrained, lower-middle income country (LMIC). According to the latest World Bank data estimated in 2022, 13.5% of residents in Bangladesh live under the poverty line and 51.6% under the lower middle-income poverty rate [8]. Fishes are the main source of animal protein (60%) in Bangladesh. Bangladeshis, particularly those belonging in the resource-deprived poor or ultra-poor segments of the population, cannot afford costly red-meat and, thus, mainly depend on the fish as a source of animal protein. However, regardless of class, fish (62.6 g/day per person) is the third most commonly consumed food in Bangladesh, after starch (471.3 g/day per person) and vegetables (167.3 g/day per person) [9]. In addition, gradual changes in the dietary habits of people of Bangladesh from well-cooked dishes to smoked fishes or fish barbecues have increased the risk of hLF infections.

Diseases due to hLFs are attributed to 1.8 million disability-adjusted life years (DALY) per year, and are estimated to have increased by 8.5% than previous estimate [10]. Early infections in humans are asymptomatic or mild; however, in chronic infections with a very heavy worm burden (up to 25,000 flukes), symptoms include bile duct obstruction, stone formation, cholangitis, pancreatitis, and hepatic fibrosis. Liver flukes may also cause metaplasia of the bile duct epithelium, which progresses to cholangiocarcinoma (CCA) [11–13]. Therefore, *C. sinensis* and *O. viverrini* have recently been classified as a group I biocarcinogen by the International Agency for Research on Cancer (IARC) of the World Health Organization (WHO). In East Asia, approximately 5000 CCA cases annually are attributed to *C. sinensis* infection [1, 11, 14]. On the other hand, *O. viverrini*-associated CCA has been estimated to cause 1200–1800 deaths per year [15].

Although human cases have yet to be identified in Bangladesh, adult hLFs, such as *C. sinensis* and *O. felineus*, were detected in street dogs and cats approximately 50 years ago [16] Based on a morphological analysis, we recently detected the MC of opisthorchiid flukes in a few small wild freshwater fish species [2]. Moreover, *Metorchis orientalis*, a zoonotic liver fluke affecting humans, carnivores, and aquatic birds, was found in the livers of ducks [17]. However, the presence of the MC of hLFs has yet to be validated by authentic molecular tools. In addition, molluscan vectors of hLFs have not yet been identified in Bangladesh. We herein estimated the prevalence of the MC of major hLFs in freshwater fishes marketed for human consumption from some selected areas of Bangladesh. The presence of the MC of flukes was confirmed using PCR and PCR-restriction fragment length polymorphism (RFLP) coupled with sequencing and bioinformatic analyses. We also detected developmental stages, particularly cercariae in freshwater snails (FWS) and adult flukes in ducks and the ova of hLF in dogs and cats.

## Methodology

### Study sites and sampling

The present study was conducted in two fish hubs of Bangladesh: Mymensingh (Coordinates: 24°45ʹ22.90” N and 90° 24ʹ23.26” E) and Kishoreganj (Coordinates: 24°45ʹ83” N and 90° 88ʹ33” E) (Supplementary Fig. 1). To collect fishes, a cross-sectional study was conducted between July 2018 and June 2022. We collected 325 wild-caught and cultured freshwater fishes belonging to 13 species from different local markets located in the study areas, and fishes were identified as previously described [18, 19]. The identified species were rohu (*Labeo rohita*, *n* = 47), mrigal (*Cirrhinus cirrhosus*, *n* = 22), grass carp (*Ctenopharyngodon idella*, *n* = 22), olive barb (*Puntis sarana*, *n* = 24), silver carp (*Hypophthalmichthys molitrix*, *n* = 22), orange fin labeo (*Labeo calbasu*, *n* = 21), bata (*Labeo bata*, *n* = 23), tilapia (*Oreochromis mossambicus*, *n* = 24), spotted snakehead (*Channa punctata*, *n* = 22), striped snakehead (*Channa striata*, *n* = 23), yellowtail catfish (*Pangasianodon hypophthalmus n* = 23), walking catfish (*Clarias batrachus*, *n* = 23), and stinging catfish (*Heteropneustes fossilis*, *n* = 29) (Supplementary Table 1). The sources of fishes were verified by fishermen during collection. Since FWS act as the first intermediate host, we collected and examined 8500 FWS of eight species: *Lymnaea auricularia* (*n* = 1150), *Lymnaea luteola* (*n* = 1100), *Indoplanorbis exustus* (*n* = 1020), *Thiara* spp. (*n* = 1056), *Brotia* spp. (*n* = 998), *Viviparus bengalensis* (*n* = 1050), *Physa* spp. (*n* = 1037), and *Bithynia* spp. (*n* = 1089). We also collected and examined 216 fecal samples from dogs (*n* = 116) and cats (*n* = 100) and 275 autopsied domestic ducks.

### Fish processing, artificial digestion, and recovery of MC

We collected 200 g of flesh from different regions of the body (dorsal, ventral, and caudal regions) of each fish, cut it into small pieces, and blended it using artificial gastric juice containing 0.3% pepsin (LOBA Chemie Pvt. Ltd., Mumbai, India) and 1% HCl (Merck, Darmstadt, Germany). We also collected gills and surrounding tissues from the head (~ 200 g). Tissues collected from the head and body were processed separately. Processed fish samples were digested overnight at 37 ºC under vigorous stirring following previously described procedures [2]. Digested materials were filtered through 1 mm of mesh to remove larger particles (bones and undigested materials), and then washed extensively with normal saline. The sediment obtained was centrifuged at 800 × *g* for 5 min, the pellet was re-suspended in phosphate-buffered saline (PBS) to a total volume of 15 ml, and 0.15 ml of the suspension was examined. Metacercariae were identified by previously reported keys and descriptions [2]. Each sample was examined at least in triplicate. The average number of MC was multiplied by 100 to estimate the total number of MC present in 100 g of fishes and kept at -20 ºC for later analyses. We also collected, processed, and examined the fins and scales of fishes separately.

### Vector snail identification

Snail species and habitats were recorded during collection. The shedding of cercariae was induced by exposing snails to light as previously described [20, 21]. Briefly, each snail was kept in separate glass test tubes, exposed to sunlight or a light source mimicking sunlight (550 lm) for 3 h, and observed. A drop of water containing cercariae was examined under a light microscope by adding 2% iodine solution. Snails shedding *Pleurolophocercus* (opisthorchiid) cercariae (PC) were recorded. Snails that did not shed cercariae were crushed and tissue fluid was examined.

### Coprological investigation

Approximately 5–10 g of freshly voided fecal samples were collected from each dog or cat in a separate specimen vial and preserved with 10% formalin. Fecal samples were examined using the sedimentation method. Briefly, 10 g of feces was added to normal saline and a uniform suspension was prepared, sieved, and left for half an hour. The supernatant was discarded and sediments was washed in the same manner until the suspension became clear. Sediment from each sample was examined in triplicate. Opisthorchiid eggs were identified by their characteristic morphological features [22].

### Collection and post-mortem examination of avian reservoir hosts

Ducks (*n* = 275) were euthanized, and the liver and gall bladder were collected. Gall bladders were opened and examined. Livers were cut into small pieces and placed individually into a separate jar containing PBS and kept for 2 h. Then the liver tissues were removed, washings were kept again for 30 min to settle down the parasites. Supernatant was removed and the sediment was examined to isolate the parasite. Adult flukes were collected and identified by preparing permanent slides by previously reported keys and descriptions [17, 22].

### DNA extraction

The genomic DNA (gDNA) of MC, cercariae, and adult flukes was extracted using the QIAamp DNA Mini Kit (Qiagen, Hilden, Germany) following the manufacturer’s instructions. Briefly, 50 mg of samples containing MC, cercariae, or adult worms was washed extensively with 1 ml of PBS by centrifuging at 2200 × *g* for 5 min. After washing, the pellet was re-suspended in 180 µl of lysis buffer supplemented with 20 µl of proteinase K and then heated at 56 ˚C for 3 h. To facilitate lysis, samples were sonicated and centrifuged at 16,900 × *g* for 5 min and the supernatant was transferred to the column. Captured DNA was eluted by adding 50 µl of elution buffer, its concentration was measured, and it was then stored at -20 ºC for further analyses. To isolate gDNA from eggs, microscopically positive samples were processed, and DNA was extracted using the FavorPrep Stool DNA isolation Mini Kit (Favorgen Biotech Corp., Michigan, USA).

### PCR assay

To validate species, the *ITS2* gene was amplified by a set of primers, which detect both *C. sinensis* and *O. viverrini*. The PCR assay was performed in a final volume of 25 µl, consisting of 50 ng of the gDNA template, 10 pmol of RTFlukeFa: 5′-CTT GAA CGC ACA TTG CGG CC-3’ and RTFlukeRa: 5’-CAC GTT TGA GCC GAG GTC AG -3’, and 12.5 µl of One *Taq* Quick-Load 2 × Master Mix (New England BioLabs Inc., MA, UK). The PCR thermocycling profile consisted of initial denaturation at 94 °C for 15 min, followed by 35 cycles (denaturation at 94 °C for 30 s, primer annealing at 60 °C for 1 min, and primer extension at 72 °C for 2 min), with a final elongation of PCR products at 72 °C for 7 min [23]. To detect the MC of *O. felineus*, the *ITS2* gene was amplified using the primers OF-Spec-F: 5′-TGG CAT GAT TTC CCC ACG CA-3’ and OF-Spec-R: 5’-GCA TTG CCA ACA CTG GAG CC-3’ following a thermal cycle consisting of initial denaturation at 95 °C for 5 min, followed by 35 cycles (denaturation at 95 °C for 30 s, primer annealing at 59 °C for 40 s, and primer extension at 72 °C for 50 s) [24], with a final elongation of PCR products at 72 °C for 5 min. In addition, the mitochondrial *COX1* (260 bp) gene of *C. sinensis* was amplified by PCR using the newly self-designed primer sets CS_cox1F: 5′-GAT TCG TTG TTT GGT TAT GGG-3′ and CS_cox1R: 5′-CCA CAA ACC CGA TTA TCC AC-3′. The thermal cycle consisted of initial denaturation at 94 °C for 10 min, followed by 35 cycles (denaturation at 94 °C for 30 s, primer annealing at 58 °C for 1 min, and primer extension at 72 °C for 2 min), with a final elongation of PCR products at 72 °C for 5 min. To confirm MC and adult *M. orientalis*, the *28S rRNA* (340 bp) region was amplified using the following oligonucleotide primers: forward: 5′- ACG TGA TTA CCC GCT GAA CT-3′ and reverse: 5′-GTA CTT GTT CGC TAT CGG AC-3′(self-designed). The thermal cycle consisted of initial denaturation at 94 °C for 10 min, followed by 35 cycles (denaturation at 94 °C for 30 s, primer annealing at 60 °C for 1 min, and primer extension at 72 °C for 2 min), with a final elongation of PCR products at 72 °C for 5 min. PCR products were separated by 1.5% agarose electrophoresis and visualized with ethidium bromide.

### RFLP for discriminating MC of different species of hLFs

Since the RT fluke primer set amplified amplicons of very similar sizes, PCR products were digested using a restriction enzyme (*Fau*I) to distinguish *O. viverrini* and *C. sinensis* according to previously reported procedures [23]. Briefly, PCR products were incubated in a total volume of 20 µl by adding 2 units of the restriction endonuclease *Fau*I (New England Biolabs, Massachusetts, USA) at 55 ºC for 6 h. Therefore, the digested products were electrophoresed on a 2% agarose gel and visualized by ethidium bromide.

### Sequencing

PCR products were sequenced directly using appropriate forward and reverse primers. PCR products were purified on a column (Wizard PCR-Preps, Promega) and then directly subjected to sequencing (BigDye Terminator v.3.1 cycle sequencing kit, Applied Biosystems) using an automated sequencer [PRISM3730, ABI using the respective forward and reverse primers (in separate reactions)]. Forward and reverse sequences were aligned and edited using BioEdit software. Sequences were aligned using MEGA v.10.1.8 software [25] and deposited in GenBank.

### Bioinformatic analysis

The homologies of newly retrieved sequences were searched by BLAST (https://blast.ncbi.nlm.nih.gov/Blast.cgi). The aligned sequences of *ITS2* for *C. sinensis* (Accession No: OP824857–OP824863), *COX1* for *C. sinensis* (Accession No: OP824851–OP824853), *ITS2* for *O. viverrini* (Accession No: OP824870–OP824876), *ITS2* for *O. felineus* (Accession No: LC739559–LC739564), and *28S rRNA* for *M. orientalis* (Accession No: OQ913905–OQ913909, PP188614) were submitted to GenBank. To detect mutations, sequences were aligned with the reference genes (Accession No: MK886663.2, EU926762.1, AF217094.1, and MK482055 for *O. viverrini*, *O. felineus*, *C. sinensis*, and *M. orientalis* respectively) using Clustal Ω (https://www.ebi.ac.uk/Tools/msa/clustalo/) [25]. To detect genetic relationships, phylogenetic trees were constructed using maximum likelihood (ML), neighbor-joining (NJ), and maximum parsimony (MP) methods based on the Tamura-Nei model. Confidence limits were assessed using the bootstrap procedure (1000 replicates) and other settings were obtained using default values in MEGA v.10.1.8 [25]. A 50% cut-off value was implemented for the consensus tree. The outgroup and accession numbers of the previously deposited sequences are indicated in the relevant figure legends.

### Statistical analysis

Data regarding MC and cercariae harvested from fishes and FWS, respectively, were entered into an Excel file and cross-checked by two independent researchers. Descriptive statistics were used to describe the prevalence of FZT infections. Data were statistically analyzed by SPSS software (Statistical Package for Social Sciences version 20; SPSS Inc., Chicago, Illinois, USA) using a number of parametric and non-parametric tests. The infection rate of fishes was analyzed using the *Z*-test and the loads of MC in different species were compared using the chi-squared (*χ*^2^) test. *P* < 0.05 was considered to be significant.

## Results

### Infection status of zoonotic hLFs in freshwater fishes

**Fig. 1:**
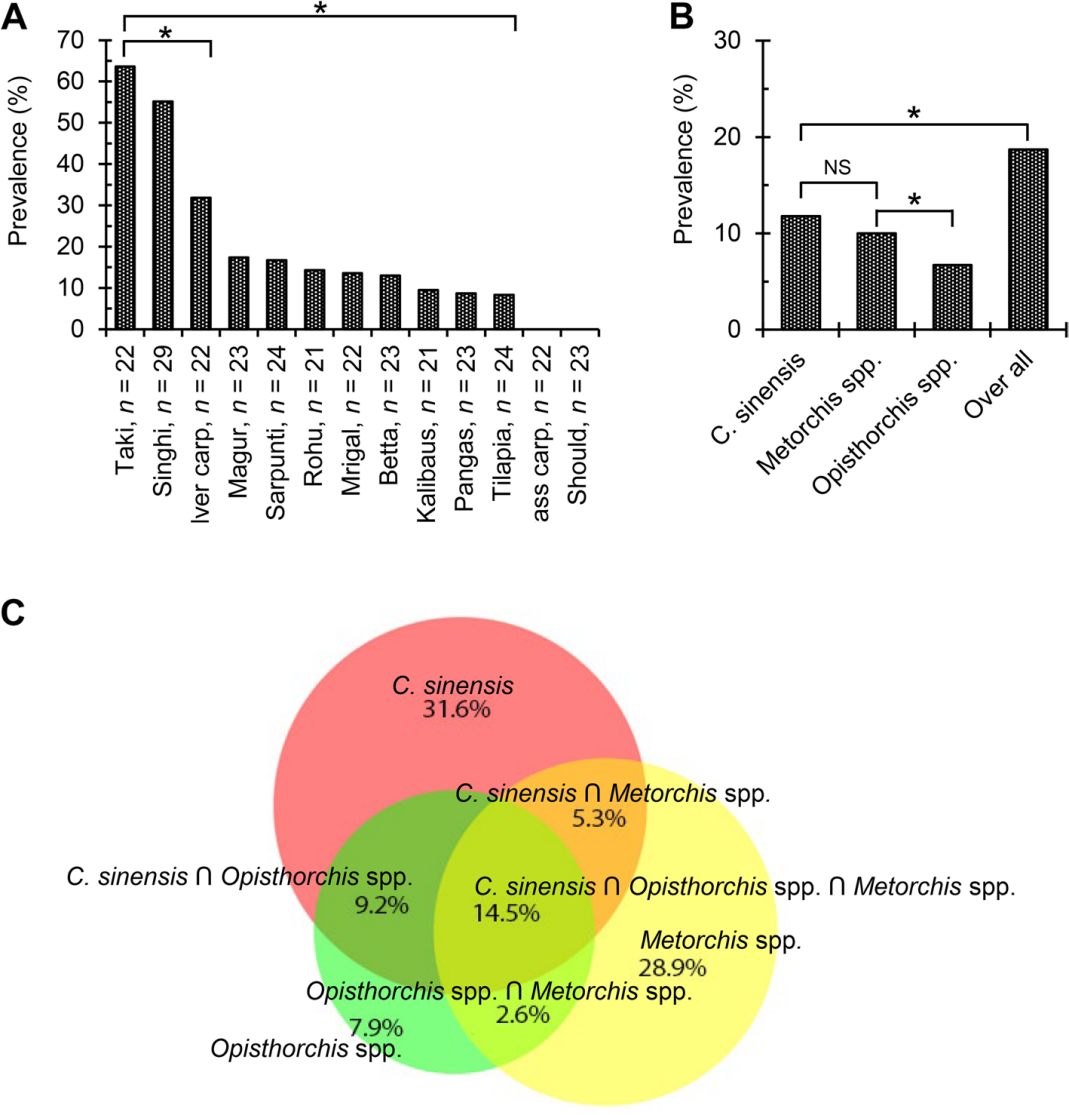
Distribution of metacercariae (MC) of human liver flukes (hLFs) in different large freshwater fishes. ^*^P < 0.05. **A** Prevalence of the MC of hLFs in different fishes. ^*^P < 0.05. **B** Overall prevalence of the MC of different hLFs. **C** Relative distribution of the MC of hLFs

In the present study, 325 lFWF, representing 13 different species, were collected from various local markets and examined for hLF infections. Of the 13 species examined, rohu (*L. rohita*), mrigal (*C. cirrhosus*), olive barb (*P. sarana*), silver carp (*H. molitrix*), orange fin labeo (*L. calbasu*), bata (*L. bata*), tilapia (*O. mossambicus*), spotted snakehead (*C. punctata*), yellowtail catfish (*P. hypophthalmus*), walking catfish (*C. batrachus*), and stinging catfish (*H. fossilis*) were infected with the MC of different hLFs. Any types of MC were not detected in grass carp (*C. idella*) and striped snakehead (*C. striata*) (Supplementary Fig. 2A). The average prevalence of the MC of hLFs was 18.7% in lFWF with a mean intensity of 137.4 ± 149.8 MC per 100 g of fish. However, significantly higher prevalence (*P* < 0.05) was detected in several fish species, such as *C. punctata* (63.6%), *H. fossilis* (55.1%), and *H. molitrix* (31.8%), but the lowest prevalence was found in *O. mossambicus* (8.3%). In addition, the mean load of MC per 100 g of fish was the highest (*P* < 0.05) in *L. calbasu* (319.5 ± 0.0) and the lowest in *L. rohita* (11.0 ± 2.6) (Fig. 1A; Table 1). We tentatively identified the MC of opisthorchiid flukes based on morphological and morphometrical features as previously reported [2]. The morphological analysis revealed three types of MC: *C. sinensis*, *Opisthorchis* spp., and *Metorchis* spp. The MC of *C. sinensis* were elliptical and measured 0.15–0.17 × 0.13–0.15 mm in size, with oral and ventral suckers of similar sizes, brownish pigment granules, and an O-shaped excretory bladder. Both MC of *C*. *sinensis* and *Opisthorchis* spp. shared common features; however, the MC of *Opisthorchis* spp. were slightly larger (0.19–0.25 × 0.15–0.22 mm). The MC of *Metorchis* spp. was almost circular (0.17 ± 0.06 × 0.17 ± 0.05 mm) and characterized by the presence of a clearly visible double-layered cyst wall (Supplementary Fig. 2B).

**Table 1:** Infection rate and load of MC in large freshwater fishes

Fish species	Type of fishes	Fishes examined (Infected fishes)	Load of MC/100 g		FZTs
			Range	Mean ± SD	
*Channa punctata* (Spotted snakehead, *Taki*)	C & WC	22 (14)	11-2616	306.3^a^ ± 403.4	*C. sinensis, Opisthorchis* spp., *Metorchis* spp.
*Heteropneustes fossilis* (Stinging catfish, *Singhi*)	C & WC	29 (16)	19–837	303.0^a^ ± 254.4	*C. sinensis, Opisthorchis* spp., *Metorchis* spp.
*Hypophthalmichthys molitrix* (Silver carp)	C	22 (07)	11–833	232.0^a^ ± 303.3	*Metorchis* spp.
*Puntis sarana* (Olive barb, *Sarpunti*)	C & WC	24 (05)	9-558	265.7^a^ ± 289.4	*C. sinensis, Opisthorchis* spp., *Metorchis* spp.
*Clarias batrachus* (Walking catfish, *Magur*)	C & WC	23 (04)	100–220	142.3^b^ ± 53.1	*Metorchis* spp.
*Labeo rohita* (Rohu)	C & WC	47 (03)	9–14	11.0^b^ ± 2.6	*Opisthorchis* spp.
*Cirrhinus cirrhosis* (Mrigal)	C & WC	22 (03)	21–36	27.0^b^ ± 7.9	*Opisthorchis* spp.
*Labeo bata* (Bata)	C & WC	23 (03)	8–49	30.0^b^ ± 20.6	*Opisthorchis* spp.
*Labeo calbasu* (Orange fin labeo, *Kalibaus*)	C & WC	21 (02)	192–447	319.5^a^ ± 0.0	*C. sinensis / Opisthorchis* spp.
*Pangasianodon hypophthalmus* (Yellowtail catfish, *Pangas*)	C	23 (02)	19-24.5	21.7^b^ ± 0.0	*C. sinensis, Opisthorchis* spp., *Metorchis* spp.
*Oreochromis mossambicus*, (Tilapia)	C	24 (02)	44–212	128.0^b^ ± 0.0	*Metorchis* spp.
*Ctenopharyngodon idella* (Grass carp)	C	22 (00)	0–0	0.0 ± 0.0	None
*Channa striata* (Striped snakehead, *Shoul*)	C & WC	23 (00)	0–0	0.0 ± 0.0	None

Values with different superscripts (e.g., a and b) in the same column are statically significant (*P* < 0.05). *C* Cultured, *WC *Wild caught, *MC* Metacercariae, *FZTs* Fish-borne zoonotic trematodes

### Prevalence and distribution of MC of hLFs in infected fishes

Among the isolated MC of hLFs, the highest (*P* < 0.05) prevalence was found in *C. sinensis* (11.8%) followed by *Metorchis* spp. (10.0%) and *Opisthorchis* spp. (6.7%) (Fig. 1B). Our Venn diagram study revealed that infection rates with the MC of *C. sinensis*, *Opisthorchis* spp., and *Metorchis* spp. were 31.5%, 7.8%, and 28.9%, respectively. Mixed infections with *C. sinensis* and *Opisthorchis* spp., with *C. sinensis* and *Metorchiss* spp., and with *Opisthorchis* spp. and *Metorchiss* spp. were detected in 9.2%, 5.2%, and 2.6% of fishes, respectively. Furthermore, a mixed infection with three liver flukes was observed in 14.4% of fishes (Fig. 1C).

To estimate the distribution of MC in fishes, we processed the head and body of fishes separately. The results obtained revealed that the MC of hLFs were distributed in both the head and body in a similar manner. However, the infection rate was slightly higher in the flesh (9.8%) than in the head (7.4%) of fishes (*P* > 0.05). Furthermore, we collected the scales of scaly fishes, processed them separately, and did not find MC in the scales or fins of fishes.

### Relative distribution of hLFs in wild-caught and cultured fishes

In Bangladesh, wild-caught and cultured fishes are both marketed for human consumption. Therefore, a comparable number of wild-caught (*n* = 126) and cultured fishes (*n* = 199) were purchased, processed, and examined. We found that wild-caught and cultured fishes were both almost equally infected with the MC of hLFs. However, the prevalence of hLFs was slightly (*P* > 0.05) higher in cultured fishes (19.6%) than in wild-caught fishes (17.5%) (Supplementary Fig. 3).

### Validation of different species of liver flukes by PCR- RFLP

**Fig. 2:**
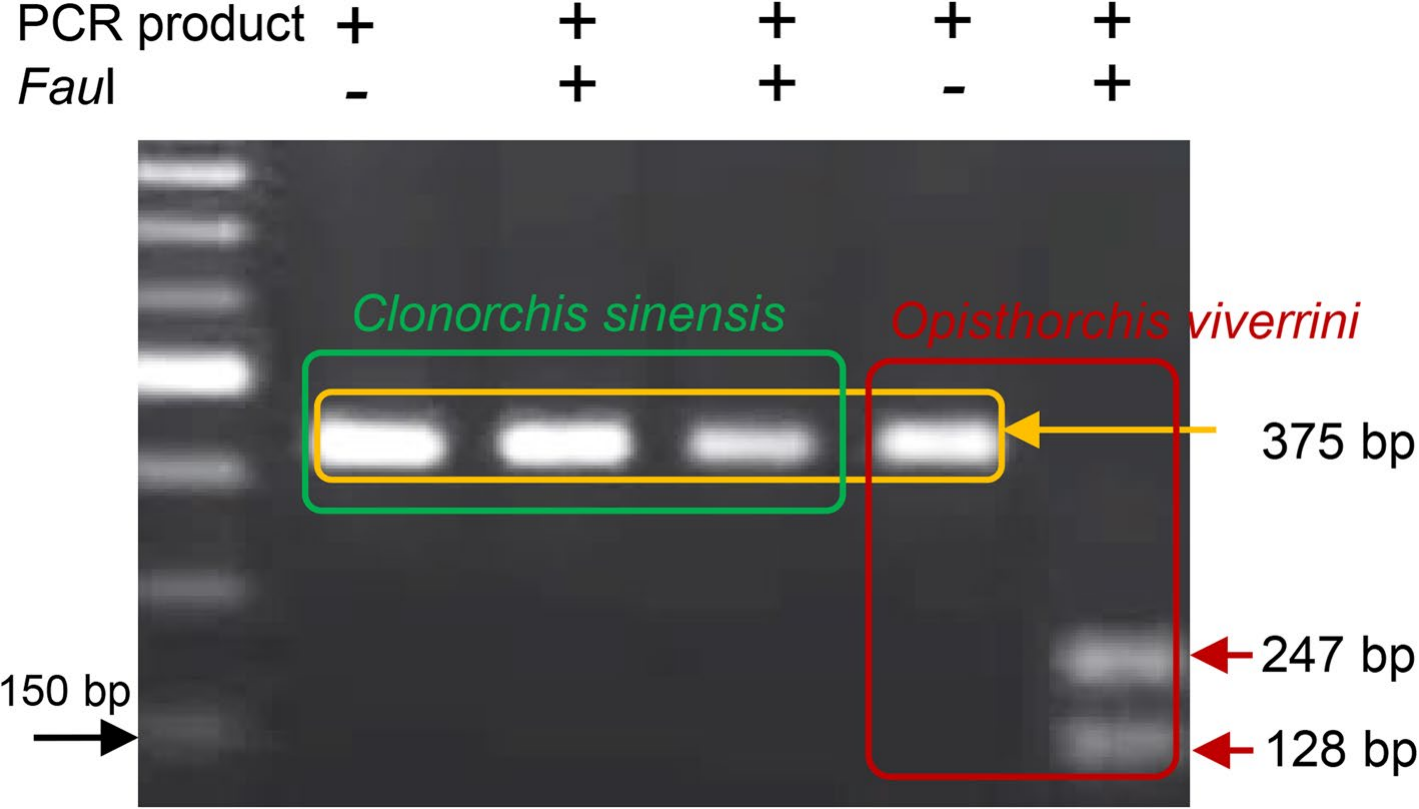
PCR-RFLP for the confirmation of *Clonorchis sinensis* and *Opisthorchis viverrini*. *ITS2*-PCR products of *O*. *viverrini* were digested with the *Fau*I restriction enzyme. Amplicons of 247 and 129 bp from *ITS2*-PCR products of *O*. *viverrini* were generated after digestion

To overcome the limitations of microscopic examination of MC, we utilized previously developed PCR coupled with other molecular tools to confirm species [26, 27]. We amplified the *ITS2* gene, which amplified both *C. sinensis* and *O. viverrini* with amplicon sizes of 381 and 375 bp, respectively. *Fau*I digested the *O. viverrini*-specific band, while the *C. sinensis*-specific band remained undigested. By employing PCR-RFLP, we confirmed the presence of the MC of both *C. sinensis* and *O. viverrini* in Bangladesh (Fig. 2). Furthermore, a self-designed primer of the *COX1* gene of *C. sinensis* was used in the present study, and we found amplicons of the expected size (260 bp) by PCR amplification. To identify the MC of *O. felineus*, PCR was conducted using species-specific primers and an amplicon of the expected size (207 bp) was detected. To confirm the MC of *Metorchis* spp., amplification of the *28S rRNA* gene was performed using a species-specific primer set, and we detected an amplicon of the expected size (400 bp) by PCR (data not shown).

### Genetic analysis of the MC of hLFs in fishes

**Fig. 3:**
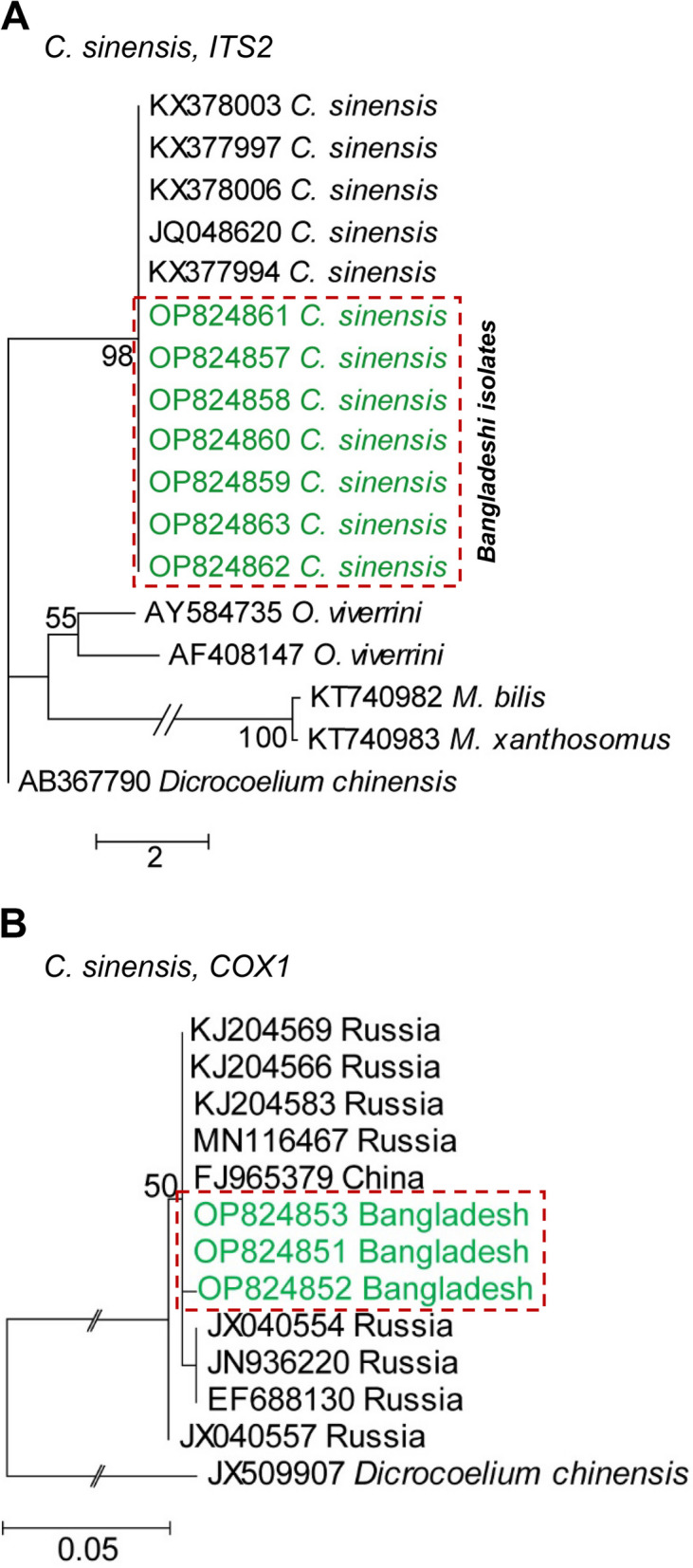
Phylogenetic analysis of *Clonorchis sinensis.* **A** Phylogenetic relationships of *C. sinensis* based on the mitochondrial gene *ITS2* inferred from a maximum likelihood (ML) tree and *Dicrocoelium chinensis* (GenBank accession nos. AB367790) served as the outgroup. **B** Phylogenetic relationships of *C. sinensis* based on the mitochondrial gene *COX1* inferred from the ML tree and *Dicrocoelium chinensis* (GenBank accession nos. JX509907) served as the outgroup. Newly sequenced isolates are colored in green. The scale bar indicates the expected number of substitutions per site

**Fig. 4:**
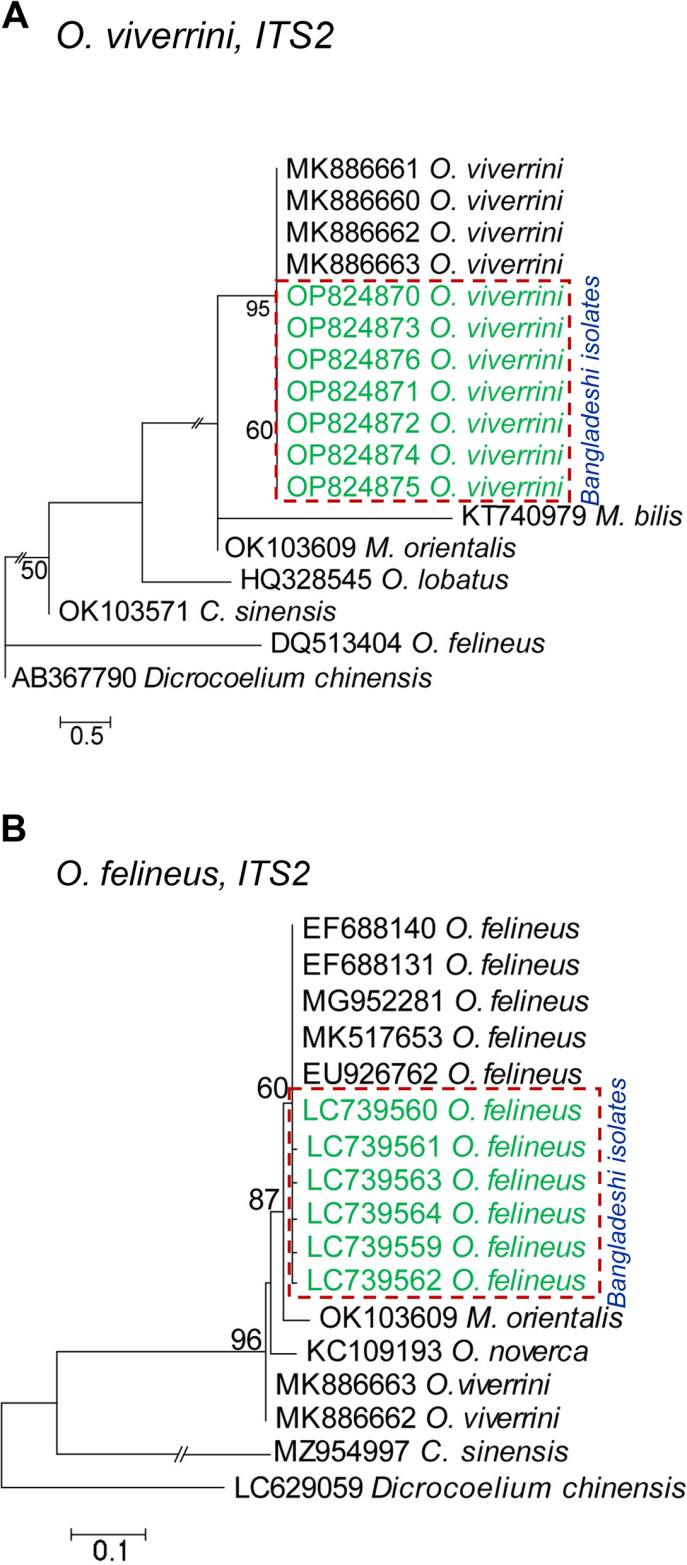
Phylogenetic analysis of *Opisthorchis viverrini* and *O. felineus*. **A** Phylogenetic relationships of *O. viverrini* based on the mitochondrial gene *ITS2* inferred from a maximum likelihood (ML) tree and *Dicrocoelium chinensis* (GenBank accession no. AB367790) served as the outgroup. **B** Phylogenetic relationships of *O. felineus* based on the ribosomal gene *ITS2* inferred from a ML tree and *Dicrocoelium chinensis* (GenBank accession no. LC629059) served as the outgroup. Newly sequenced isolates are colored in green. The scale bar indicates the expected number of substitutions per site

In the genetic analysis of *C. sinensis*, PCR products were sequenced and retrieved *ITS2* sequences were compared with the already deposited *ITS2* sequences of *C. sinensis*. A BLAST search provided homologous sequences and those with up to 99.4–100% identity were selected for a phylogenetic analysis. The newly retrieved sequences were aligned with the reference gene (Accession number: KX377994), point nucleotide mutations in the *ITS2* gene of *C. sinensis* were detected at positions 112, 199, 202, 225, 286, and 296, and only transversion mutations were observed. To detect genetic relationships, phylogenetic analyses using the NJ, ML, and MP methods were performed and all three methods showed similarity. Phylogenetic analyses revealed that Bangladeshi isolates belonged to a distinct cluster with the reference sequences (Accession number: KX378003, KX377997, KX378006, JQ048620, and KX377994) of *C. sinensis*, confirming further the species as *C. sinensis* (Fig. 3A). To reinforce this result, the *COX1* PCR products of positive samples were also sequenced, homologous sequences were searched by BLAST, and newly retrieved sequences showed very high identities (100%) with several sequences (Accession numbers: KJ204569, KJ204566, KJ204583, MN116467, and FJ965379) of *C. sinensis* reported from Russia and China. A phylogram was constructed using the sequences of the present study and those deposited in GenBank from other countries. The constructed phylogram showed that the sequences of the present study formed a closely related cluster with those reported from China and Vladivostok of Russia. However, the new sequences of Bangladeshi isolates were slightly separate from those reported from other parts of Russia (Fig. 3B). Similarly, positive samples of the *O. viverrini*-specific *ITS2* gene were amplified and sequenced. The new sequences of the *ITS2* gene of *O. viverrini* were aligned with the reference gene (Accession number: MK886663), and we detected point mutations at positions 21, 22, 97, 100, 104, 184, 231, 241, and 244. Transversion (6) and transition (3) mutations were both detected. Through a phylogenetic analysis, we showed that *O. viverrini* formed a distinct cluster with the reference sequences (Accession number: MK886660–MK886663) of *O. viverrini*, confirming that the isolate was *O. viverrini* (Fig. 4A).

The PCR products of *O. felineus* flukes were also sequenced and retrieved *ITS2* sequences were compared with the already deposited *ITS2* sequences of *O. felineus*. In a BLAST search, homologous sequences were found and sequences with up to 99–100% identity were selected for a phylogenetic analysis. Newly retrieved sequences were aligned with the reference gene (Accession number: EU926762) and six transversion mutations in the *ITS2* gene of *O. felineus* at positions 126, 130, 133, 146, 148, and 155 were detected. The phylogenetic analysis revealed that these isolates belonged to a distinct cluster with the reference sequences (Accession number: EU926762, EF688131, EF688140, MG952281, and MK517653) of *O. felineus*, validating the species as *O. felineus* (Fig. 4B).

**Fig. 5:**
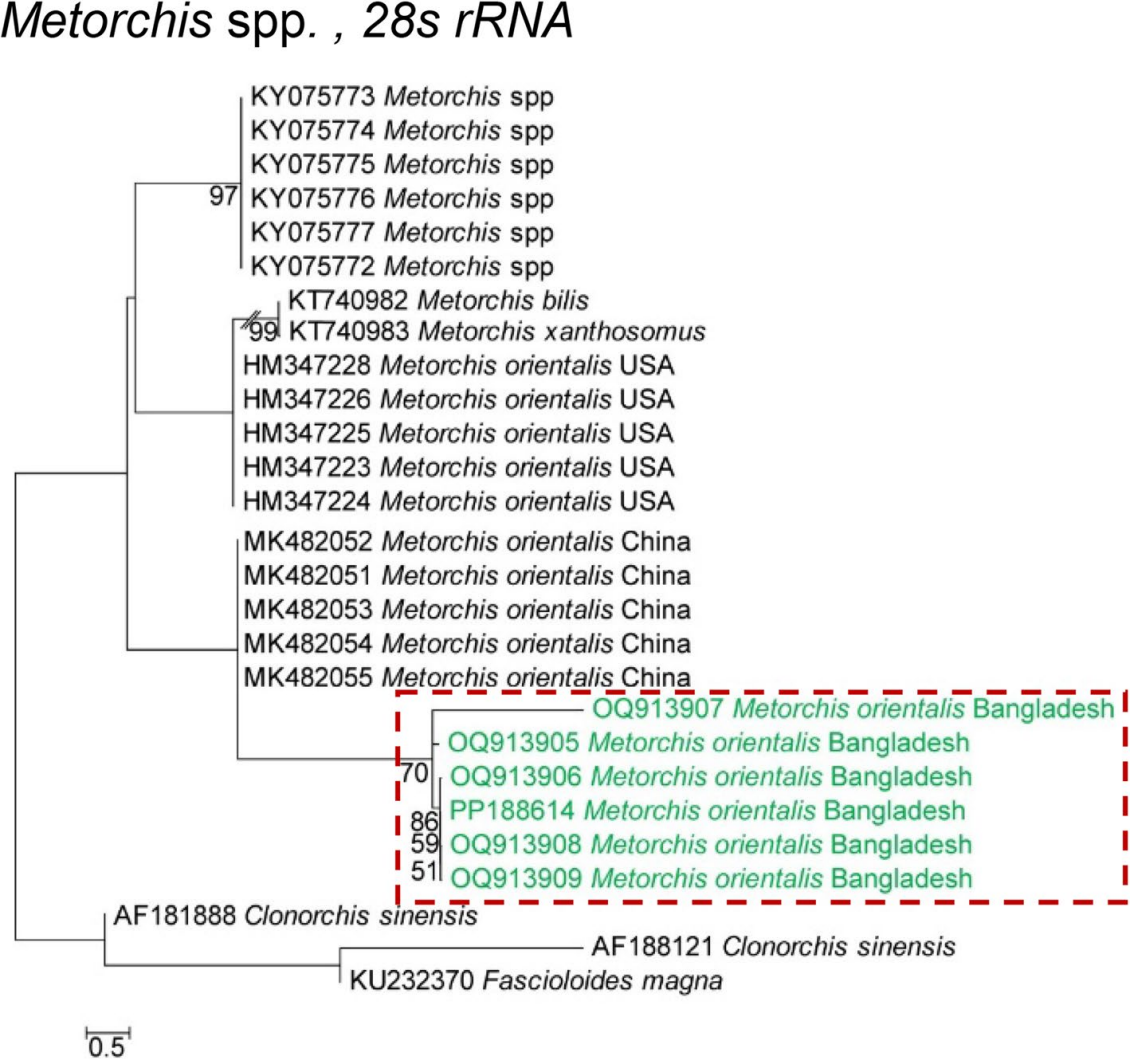
Phylogenetic analysis of *Metorchis orientalis* based on the *28S rRNA* gene inferred from a maximum likelihood (ML) tree and *Fsciolodes magna* (GenBank accession nos. KU232370) served as the outgroup. Newly sequenced isolates are colored in green. The scale bar indicates the expected number of substitutions per site

Amplicons of *28S rRNA* derived from *M. orientalis* were also sequenced and identical sequences were searched. Through a BLAST search, identical sequences with higher homology (> 99%) were detected. A phylogenetic analysis showed that *M. orientalis* formed a distinct cluster with the reference sequences (Accession number: MK482052–MK482055) of *M. orientalis*, confirming the isolate as *M. orientalis* (Fig. 5).

### Confirmation of vector snails and reservoirs

**Fig. 6:**
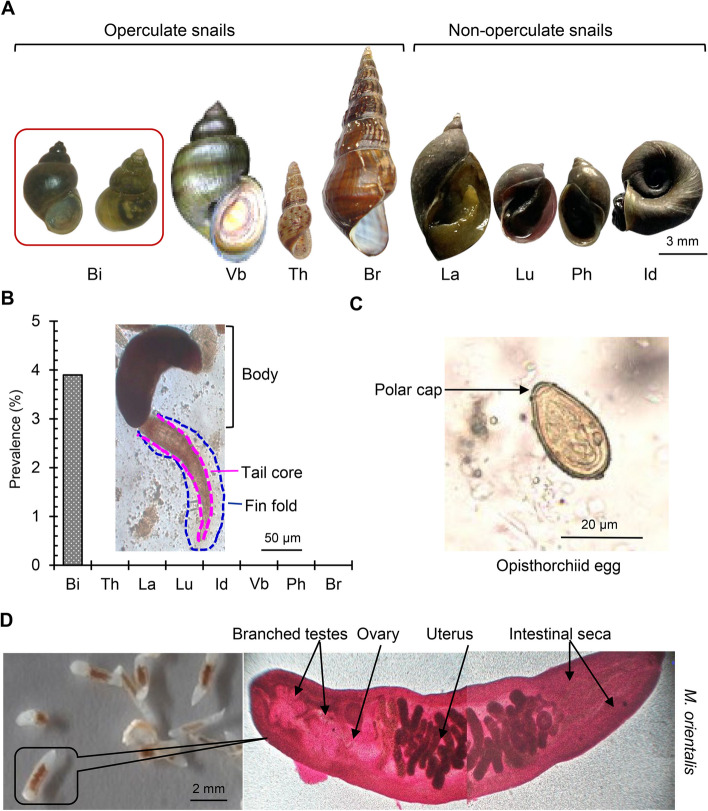
Vector, cercariae, and ova of human liver flukes and adult *Metorchis orientalis*. **A** Snails were collected and examined for *Pleurolophocercus* cercariae. **B** Vector snail preference and diversity of opisthorchiid flukes in Bangladesh. Inset: *Pleurolophocercus* cercariae were morphologically identified by the presence of a fin fold in their tail region. **C** The morphology and internal structure of opisthorchiid eggs recovered from dog and cat feces. **D** Adult parasites of *M. orientalis* isolated from domestic ducks. Bi, *Bithynia* spp; Vb, *Viviparus bengalensis*; Th, *Thiara* spp.; Br, *Brotia* spp.; La, *Lymnaea auricularia*; Lu, *Lymnaea luteola*; Ph, *Physa* spp. Id, *Idoplaorbis exustus*

Among the snails examined, the shedding of PC was only detected in *Bithynia* spp. and 3.9% (42 out of 1089) of bithynid snails were infected. *Bithynia* snails were found in both natural and man-made water bodies and were attached to submerged hard objects, such as stones, boats, logs, and the leaves of aquatic plants. *Bithynia* spp. are operculate snails. They were globose in shape and measure 12–15 × 5–7 mm in size. They were dextral with a pointed apex, brownish in color, and had a median suture (Fig. 6A, B). The cercariae (PC) were morphologically identified by the presence of a fin fold in the tail region (Fig. 6B). To validate the morphometric identification of PC, we isolated DNA and performed a PCR analysis followed by sequencing, which confirmed opisthorchiid cercariae.

In a coprological examination, we identified the eggs of *Clonorchis*/*Opisthorchis* spp. in the fecal samples of dogs (4.3%, 5 out of 116) and cats (6%, 6 out of 100). Morphologically, these eggs were light yellowish in color, and 29–34 × 13–17 μm in size, and contained a well-developed miracidium. The operculum was large and had a small hook-like structure at the rear end. The operculum was fit to the rim of the shell, giving rise to a bulb-shaped appearance (Fig. 6C). To confirm the tentative identification, we isolated DNA from eggs and subjected it to a PCR analysis followed by sequencing. Our molecular study on eggs confirmed that the species was of an opisthorchiid fluke origin. Adult *Metorchis* spp. were isolated from duck livers and identified based on their morphological features. Morphologically, the parasites were yellowish to brown in color and the tegument was covered with spines. Flukes were 2.5–6.0 mm long and 0.8–1.2 mm wide. The oral and ventral suckers were similar in size. The vitellaria was granular and bunchy, lying on both sides of the body. The tubular uterus containing eggs twisted through the ovary towards the gonopore, which was located at the anterior ventral sucker. The ovary was oval in shape and located in front of the testes. The testes were tandem and located at the posterior fourth part of the body (Fig. 6D). We found that 41.1% (113 out of 275) ducks were infected with *M. orientalis*. After their tentative identification, PCR amplification was performed and a DNA fragment of the *28S rRNA* gene of the expected size (400 bp) was amplified from adult flukes, confirming the presence of *M. orientalis* in ducks. We sequenced PCR products and performed a bioinformatic analysis for further confirmation, which got similar results to those retrieved from MC of *M*. *orientalis*.

## Discussion

Liver flukes, particularly *C. sinensis*, *O. viverrini*, *O. felineus*, and *Metorchis* spp., cause fatal liver cirrhosis and are regarded as an emerging public health issue globally, particularly in Southeast Asia. The flukes live in the hepatobiliary system and are considered to be carcinogenic agents because they are involved with the development of CCA in humans [11]. Despite tremendous efforts to control and mitigate hLF infections, the burden of food-borne trematode infections is increasing [10], and being reported in new areas. In this modern era when food security is given the first priority, a new segment of the global population is being exposed to food-borne zoonotic hLF infections [1, 2]. Since these flukes are essentially borne by snails and fishes, the most effective preventative strategy is to control or manage the spread of infectious agents through molluscan and fish intermediate hosts. We herein surveyed the MC of hLFs in common and popular lFWF. Also, we validated hLF species along with genetic analyses and the confirmation of molluscan vectors and reservoirs.

The results obtained showed that 18.7% lFWF were infected with the MC of hLFs. We very recently conducted a survey to detect the MC of FZTs in a few wild-caught small indigenous fishes (SIF), which revealed that FZTs, including hLFs, were prevalent in 66.2% of the selected SIF [2]. Although hLF infections have yet to be reported in humans living in Bangladesh, many human cases had already been reported in Southeast and Fareast countries, namely Thailand, China, Japan, Republic of Korea, Lao PDR, Cambodia, Vietnam, and Myanmar [28]. We detected the MC of *C. sinensis*, *O. viverrini*, *O. felineus*, and *M. orientalis* in 11 species of wild and cultured fishes belonging to different families, namely Cyprinidae, Heteropneustidae, Pangasiidae, Channidae, Clarridae, and Cichlidae, suggesting that they play significant roles as second intermediate hosts to complete the life-cycle of hLFs in Bangladesh. More than 100 fish species have been identified as second intermediate hosts of fish-borne trematodiosis. Among them, commonly consumed cyprinid fishes play a central role and act as the primary source of human infections in many Southeast Asian countries [29]. The large number of fish species reported to be infected with the MC of liver flukes implies the high adaptability of these flukes in nature (WHO, 1995). Along with cyprinid fishes, the MC of opisthorchiid flukes have been detected in Pangasiidae, Channidae [30] as well as in *Tilapia* spp. (family: Cichlidae) in Vietnam [31], suggesting that these fishes, other than cyprinids, also act as the second intermediate host of hLFs.

Since the fish species examined herein are very widely distributed in different natural and man-made water bodies in Bangladesh, they may contribute to human and animal infections. Among hLF infections, *C. sinensis* is the most important and significant species due to its public health impact. Existing endemic areas of clonorchiosis include China, Republic of Korea, North Vietnam, and Far East Russia [12]. *C. sinensis* infects not only cyprinids, but also an array of other fish families [28, 32] and 132 species of fishes (including 71 cyprinids) belonging to 11 families have been listed as second intermediate hosts with a prevalence ranging up to 95% in China [7]. In Republic of Korea, *Pungtungia herzi*, *Zacco platypus*, *Carassius auratus*, and *Pseudogobio esocinus* fishes were identified as second intermediate hosts of *C. sinensis* with prevalence rates of 13.5%, 12.9%, 10.8%, and 8.3%, respectively [33]. On the other hand, *O. viverrini* preferentially infects cyprinoid fishes. In the greater Mekong sub-region of Southeast Asia, more than 40 species of cyprinids were infected with *O. viverrini* with an extremely variable prevalence ranging between 2.1% and 100% [32] and 11 species of fishes have been identified as second intermediate hosts of *O. viverrini* in Cambodia with an average load of 74.6 MC per fish [34]. The Phu Yen province of Vietnam is regarded as a ‘hot spot’ for the parasite, where the prevalence of *O. viverrini* was 10–29% in the crucian carp and *C. carassius* [35]. In Thailand, five species of fishes, namely, *Henicorhynchus siamensis*, *Cyclocheilichtys* spp., *Hampala* spp., *Systomus* spp., and *Barbonymus goniatus*, were reported to be infected with *O. viverrini* MC [36]. Variations in infections by and the burden of MC among previous studies may be attributed to differences in the species of fishes, water reservoirs, fish biodiversity, the availability of vector snails and reservoir hosts, and snail-fish interactions as well as waste management, particularly the disposal of human waste and cropping patterns.

The present study suggests that wild and cultured lFWF are both equally hazardous for human health. In natural water bodies and aquaculture systems, the main source of infection and transmission include the contamination of water bodies with eggs from infected hosts, i.e., humans, cats, dogs, pigs, and other fish-eating mammals and birds. In Bangladesh, reservoir hosts roam freely and have easy access to natural water bodies and the vicinity of aquaculture systems. They defecate near the banks of rivers, ponds, and canals, and feces is washed into the water by rain. Additionally, ponds become contaminated through their use by animals and occasionally by human fecal waste as pond fertilizer and through run-off water from pond banks and adjoining fields; therefore, the risk of infection with the MC of hLFs is similar in fishes harvested from natural and man-made water bodies.

Through a well-recognized pepsin digestion method, we revealed that the MC of hLFs were equally distributed in both the gills and flesh of fishes, suggesting that the entire fish is equally important for infection. However, the gills are generally not used for human consumption and are discarded during processing, but are picked up by fish-eating mammals and birds, namely, dogs, cats, foxes, and ducks, which strongly contributes to the transmission of infection to domestic and peri-domestic reservoirs. The present study proved that the FWS-fish-domestic/peri-domestic reservoir cycle of hLFs is competent enough for the survival and existence of the flukes in nature even without infecting humans. In some studies, MC were mostly recovered from the scales, fins, and tails [37]; however, we did not detect MC in the scales and fins of fishes in the present study, which may have been due to variations in the method used to collect scales. We removed and processed scales without skin or underlying tissues. However, in some territories, scales were removed with attached skin during processing.

Regarding the molecular validation of species, we used a previously standardized *ITS2* multiplex PCR, which simultaneously detects *C. sinensis* (381 bp) and *O. viverrini* (375 bp); therefore, *C. sinensis* and *O. viverrini* amplicons obtained by electrophoresis were almost indistinguishable. We digested PCR products with *Fau*I and detected both degraded and non-degraded amplicons, which confirmed the presence of both *C. sinensis* and *O. viverrini*. The targeted part of the *ITS2* gene from *O. viverrini* contains a site that is cleaved by *Fau*I and, thus, the enzyme produces two fragments. However, since the same segment of the *ITS2* gene from *C. sinensis* did not have the *Fau*I cleavage site, the amplicons retrieved from *C. sinensis* remained unaffected. Various types of PCR-RFLP have been developed to validate the species of hLFs and are being utilized in different countries. By employing a PCR-RFLP analysis of the *ITS2* region, a cross-sectional study was initially conducted in 2009 to identify *C*. *sinensis* infection among a population of Na-Yao villagers in Thailand. The developmental stages, eggs, and adult parasites of *C*. *sinensis* were detected in the areas examined [38].

Additionally, through bioinformatic analyses, newly retrieved sequences showed few point mutations when compared with reference sequences. Reference sequences were from other countries; therefore, a few point mutations were expected. A phylogenetic analysis revealed that our sequences produced a distinct cluster only with those of *O. viverrini* or *C. sinensis*, re-confirming the validation of the species of flukes. Moreover, using self-designed, species-specific primer sets, the amplification of the *COX1* gene of *C. sinensis* was also successful. Similarly, sequencing and subsequent bioinformatic analyses revealed that the newly retrieved sequences had the highest identity with *C. sinensis* or *O. viverrini*, providing unambiguous proof for the confirmation of the species.

A *COX1*-based phylogram showed that the Bangladeshi isolates produced a very closely related cluster with the isolates from Russia and China. Although a few parts of China are nearer to Bangladesh, but far from Russia. Russia is a neighboring country of China, sharing a long common border. Since Bangladesh has a strong business relationship with China and imports many goods, including fishes, the introduction of hLF-like opisthorchiid flukes to Bangladesh from China and its neighboring countries is not unlikely. The MC of *O. felineus* is indistinguishable from that of *O. viverrini.* Therefore, a set of species-specific primers was used that amplified the *ITS2* gene of *O. felineus*. The primer did not show any cross-reactivity [24], confirming the presence of *O. felineus* in the study area. In addition to PCR, sequencing and bioinformatic analyses showed that the newly retrieved sequences had the highest similarity with that of *O. felineus* deposited previously in GenBank, which reconfirmed the presence of the fluke in Bangladesh. A phylogenetic tree generated with the new sequences also showed a distinct cluster only with that of *O. felineus*, providing unambiguous proof for the confirmation of species. By applying multiple molecular tools, we unambiguously demonstrated that all three important species of major hLFs, such as *O*. *viverrini*, *C*. *sinensis*, and *O*. *felineus*, were prevalent in the study area. The simultaneous occurrence of the three species of liver flukes in Bangladesh was not surprising because fishes and fish products are both imported from neighboring endemic countries, such as Vietnam, Myanmar, Thailand, China, and India. In addition, there are frequent movements of people to these endemic countries for trade, tourism, and other purposes. Furthermore, there was a large refugee settlement to Bangladesh from Myanmar recently, which is yet to be investigated, but may be another source for the transmission of these pathogens from a recognized endemic area to Bangladesh.

The presence of *M. orientalis* was confirmed by a self-designed primer set targeting the *28S rRNA* gene. To reinforce the present results, PCR products were also sequenced and the sequences retrieved showed very high homology with those already deposited in GenBank for *M. orientalis*, further validating the presence of *M. orientalis* in Bangladesh. This fluke has a wide host range and may infect various fish-eating mammals, aquatic birds, and humans. Water bodies are co-occupied by snails, fishes, and aquatic birds; therefore, the presence of *M. orientalis* is not surprising but human cases have yet to be reported.

The present study recorded PC from snails belonging to *Bithynia* spp., but not from other snail species, suggesting that only bithynid snails acted as the first intermediate host of opisthorchiid flukes in Bangladesh. To the best of our knowledge, this is the first study to investigate the molluscan intermediate hosts of opisthorchiid flukes in Bangladesh. Trematodes of the families Heterophyidae, Opisthorchiidae, and Cryptogonimidae produce PC [39]. By employing molecular tools, we confirmed the PC of opisthorchiid flukes from bithynid snails. Several operculate snails, such as *Bithynia* spp., *Thaira* spp., and *Viviparus* spp., have been reported to act as the first intermediate hosts of opisthorchiid flukes [22]. A previous study reported that the prevalence of cercarial infection in bithyniid snails in Thailand was 8.4%, and found that *Bithynia siamensis* harbored seven different types of cercariae, including PC [40]. In Lao PDR, PC (0.9%) were detected in *Bithynia siamensis* snails [41]. In India, virgulate and *xiphidiocercous* cercariae were detected in *Bithynia pulchella* snails, suggesting that *Bithynia* snails act as the intermediate host of various trematodes, including hLFs. In the present study, *Bithynia* spp. were mainly found to be attached to submerged hard objects, such as stones, boats, logs, and the leaves of aquatic plants. However, bithynid snails are frequently distributed in ponds, streams, canals, and paddy fields [42]. Their location may be deemed a suitable setting for cercariae because these habitats are co-occupied by both FWS and fishes. The co-existence of the first (snails) and second (fishes) intermediate hosts facilitates the survival of hLF in a particular area. After aggressing cercariae from FWS, cercariae easily enter fishes, in which they encyst into MC.

In the present study, the eggs of opisthorchiid flukes were detected in the feces of street dogs and cats, suggesting that these animals acted as reservoirs of opisthorchiid flukes in the areas examined. Canines and felines mainly act as the reservoirs of *Opisthorchis* spp. and *C. sinensis.* In a recent study, opisthorchiid eggs were detected in the feces of dogs and cats in other parts of Bangladesh [43]. Approximately 50 years ago, opisthorchiid flukes, such as *O. tenuicollis* and *O. felineus*, were recovered from wild cats and street dogs in Bangladesh [16]. The roles of dogs, cats, and foxes in the transmission cycle of hLFs have been evaluated in different countries. Intensive epidemiological surveillance conducted in Thailand, China, and Republic of Korea detected the ova of hLFs in fecal samples collected from dogs and cats [44–46]. In the study areas, dogs and cats roam freely and have easy access to natural water bodies as well as man-made fish farms. These reservoir hosts also eat raw fishes and raw fish leftovers. Their feces easily contaminate natural and man-made water bodies and, thus, plays a significant role in the transmission of hLFs and their existence in Bangladesh. In addition to canid and felid reservoirs, aquatic birds, particularly ducks, act as reservoir of *M. orientalis*. In the present study, we also isolated and identified *M. orientalis* from the livers of ducks. Ducks are the second most common and popular poultry in Bangladesh. There are approximately 66.0 million [47] ducks in Bangladesh, most of which are reared in scavenging and semi-scavenging systems. Ducks are more common in low lying marshy areas, where they have free access to paddy fields, rivers, canals, and ponds. They consume the fishes available in natural and man-made water bodies, which increases their risk of getting infection by the MC of *Metorchis* spp. Through this multifaceted study, we assume that the “FWS-FWF-duck” cycle plays a vital role in the existence of *M. orientalis* in Bangladesh.

In the present study, we only isolate adult *M. orientalis* from ducks but we could not isolate other adult flukes, including *O*. *viverrini*, *C*. *sinesis*, and *O*. *felineus*, because adult flukes can only be detected in a postmortem examination of reservoir hosts, such as street dogs, cats, and wild carnivores (e.g., foxes and jackals). At present in Bangladesh, the killing of street dogs, cats, and wild animals is strictly prohibited. Therefore, we were unable to isolate adult flukes from these reservoirs. Alternatively, we collected feces from street dogs and cats, detected eggs of the flukes by coprological examinations, and confirmed the species of liver flukes by copro-PCR. Another limitation of the present study is that we could not collect samples, particularly fish samples, from the entire country due to funding constrain. However, the socio-economic status, knowledge, attitude and practice of the people, geo-climatic condition, aquaculture and management prevailed in the selected study areas are almost similar throughout the country. In addition, Mymensingh and Kishoreganj are the main hub of fresh water fishes of the country and from these two areas caught fishes are mainly distributed to other parts of Bangladesh.

## Conclusions

The MC of hLFs are highly prevalent in both cultured and wild-caught fishes in Bangladesh. We are the first to provide molecular evidence for the occurrence of the MC of hLFs, namely, *C. sinensis*, *O. viverrini*, *O. felineus*, and *M. orientalis*, in freshwater fishes available in Bangladesh. We also confirmed the vector snails of hLFs and identified dogs, cats, and ducks as the reservoir hosts of various hLFs. People living in Bangladesh are at risk of hLF infections and the present study will facilitate the development of a sustainable control strategy against hLFs to ensure the safety of fishes and fish products.

### Supplementary Information

Additional file 1: Supplementary Figure 1: A map showing study areas.

Additional file 2: Supplementary Figure 2: Freshwater fishes studied and metacercariae recovered. (A) Large freshwater fishes infected with the metacercariae (MC) of human liver flukes (hLFs). Mrigal (*Cirrhinus cirrhosus*),rohu (*Labeo rohita*), silver carp (*Hypophthalmichthys molitrix*), grass carp (*Ctenopharyngodon idella*), orange fin labeo (*L. calbasu*), olive barb (*Puntis sarana*), bata (*L. bata*), striped snakehead (*C. striata*), spotted snakehead (*Channa punctata*), tilapia (*Oreochromis mossambicus*), walking catfish (*Clarias batrachus*), stinging catfish (*Heteropneustes fossilis*), and yellowtail catfish (*Pangasianodon hypophthalmus*) were collected and identified. Fishes within green boxes were free from infection. (B) Isolated MC of major hLFs.

Additional file 3: Supplementary Figure 3: Prevalence of metacercariae (MC) of human liver flukes (hLFs) in cultured and wild fishes. hLFs in cultured and wild fishes.

Additional file 4.

## Data Availability

All data generated or analyzed during the present study are included in the manuscript.

## Abbreviations

CCA: Cholangiocarcinoma
DALY: Disability adjusted life years
FWS: Fresh water snail
FZTs: Fish-borne zoonotic trematodesh
LFs: Human liver flukes
IARC: International Agency for Research on Cancer
lFWF: Large fresh water fish
LMiC: Lower Middle income Country
MC: Metacercariae
NTD: Neglected Tropical Diseases
RFLP-PCR: Restriction fragment length polymorphism-polymerase chain reaction
WHO: World Health Organism
PBS: Phosphate-buffered saline
PC: Pleurlophocercus cercariae

## References

[1] Anisuzzaman, Hossain MS, Hatta T, Labony SS, Kwofie KD, Kawada H (2023). Food- and vector-borne parasitic zoonoses: global burden and impacts. Adv Parasitol.

[2] Labony SS, Alim MA, Hasan MM, Hossain MS, Islam A, Alam MZ (2020). Fish-borne trematode infections in wild fishes in Bangladesh. Pathog Glob Health.

[3] Tang Z-L, Huang Y, Yu X-B (2016). Current status and perspectives of *Clonorchis sinensis* and clonorchiasis: epidemiology, pathogenesis, omics, prevention and control. Infect Dis Poverty.

[4] Dang TCT, Yajima A, Nguyen VK, Montresor A (2008). Prevalence, intensity and risk factors for clonorchiasis and possible use of questionnaires to detect individuals at risk in northern Vietnam. Trans R Soc Trop Med Hyg.

[5] Saijuntha W, Sithithaworn P, Kaitsopit N, Andrews RH, Petney TN (2014). Liver flukes: *Clonorchis* and *Opisthorchis*. Adv Exp Med Biol.

[6] Kim J-H, Choi M-H, Bae YM, Oh J-K, Lim MK, Hong S-T (2011). Correlation between discharged worms and fecal egg counts in human clonorchiasis. PLOS Negl Trop Dis.

[7] Lun Z-R, Gasser RB, Lai D-H, Li A-X, Zhu X-Q, Yu X-B, Fang Y-Y (2005). Clonorchiasis: a key foodborne zoonosis in China. Lancet Infect Dis.

[8] World Bank Group. World development indicators, Macro Poverty Outlook., 2022. https://thedocs.worldbank.org/en/doc/ab46b9f05a34cb860d84774b7bfbd77f-0500052021/related/data-bgd.pdf. Accessed 24 Mar 2024.

[9] Rifat MA, Wahab MA, Rahman MA, Nahiduzzaman M, Mamun A-A (2023). Nutritional value of the marine fish in Bangladesh and their potential to address malnutrition: a review. Heliyon.

[10] Collaborators HALE, GBD 2017 DALYs and (2018). Global, regional, and national disability-adjusted life-years (DALYs) for 359 diseases and injuries and healthy life expectancy (HALE) for 195 countries and territories, 1990–2017: a systematic analysis for the global burden of Disease Study 2017. Lancet.

[11] Bouvard V, Baan R, Straif K, Grosse Y, Secretan B, El Ghissassi F (2009). A review of human carcinogens–part B: biological agents. Lancet Oncol.

[12] Hung NM, Madsen H, Fried B (2013). Global status of fish-borne zoonotic trematodiasis in humans. Acta Parasitol.

[13] Sripa B, Kaewkes S, Intapan PM, Maleewong W, Brindley PJ (2010). Food-borne trematodiases in Southeast Asia epidemiology, pathology, clinical manifestation and control. Adv Parasitol.

[14] IARC Working Group on the Evaluation of Carcinogenic Risks to Humans (2012). Biological agents. Iarc Monogr Eval Carcinog Risks Hum.

[15] Liau MYQ, Toh EQ, Shelat VG (2023). *Opisthorchis viverrini*-Current understanding of the neglected hepatobiliary parasite. Pathogens.

[16] Shaikh H, Huq MM, Karim MJ, Munzur MK (1982). Incidence of helminth parasites of domestic and wild cats and of jackals in Bangladesh. Indian J Parasitol.

[17] Anisuzzaman, Alim MA, Islam MK, Das PM, Farjana T, Mondal MMH (2005). Avian liver fluke infection in indigenous ducks in Bangladesh: prevalence and pathology. J Bangladesh Agril Unil.

[18] Rahman AKA (2005). Freshwater fishes of Bangladesh.

[19] Talwar PK, Jhingran AG (1991). Inland fishes of India and adjacent countries.

[20] Frahm S, Anisuzzaman A, Prodjinotho UF, Vejzagić N, Verschoor A, Da Prazeres Costa C (2019). A novel cell-free method to culture *Schistosoma mansoni* from cercariae to juvenile worm stages for in vitro drug testing. PLOS Negl Trop Dis.

[21] Labony SS, Paul S, Alim MA, Hossain MS, Inoue T, Ritu SN (2022). Research note: genetic analysis, pathology, and vectors of echinostomiasis, a zoonotic helminth infection in chickens in Bangladesh. Poult Sci.

[22] Soulsby EJL (1982). Helminths, arthropods and protozoa of domesticated animals.

[23] Buathong S, Leelayoova S, Mungthin M, Ruang-Areerate T, Naaglor T, Suwannahitatorn P (2017). Molecular discrimination of *Opisthorchis*-like eggs from residents in a rural community of central Thailand. PLOS Negl Trop Dis.

[24] Bekenova AB, Smagulova A, Katokhin A, Borovikov S, Kiyan V. Molecular differential diagnosis between *Opisthorchis felineus* and *Metorchis bilis*. Adv Anim Vet Sci. 2020;8:27–32. 10.17582/journal.aavs/2020/8.s3.27.32.

[25] Tamura K, Stecher G, Peterson D, Filipski A, Kumar S (2013). MEGA6: Molecular Evolutionary Genetics Analysis version 6.0. Mol Biol Evol.

[26] Cai X-Q, Yu H-Q, Bai J-S, Tang J-D, Hu X-C, Chen D-H (2012). Development of a TaqMan based real-time PCR assay for detection of *Clonorchis sinensis* DNA in human stool samples and fishes. Parasitol Int.

[27] Rahman SMM, Bae YM, Hong S-T, Choi M-H (2011). Early detection and estimation of infection burden by real-time PCR in rats experimentally infected with *Clonorchis sinensis*. Parasitol Res.

[28] Sithithaworn P, Andrews RH, de van Nguyen, Wongsaroj T, Sinuon M, Odermatt P (2012). The current status of opisthorchiasis and clonorchiasis in the Mekong Basin. Parasitol Int.

[29] Sohn W-M (2009). Fish-borne zoonotic trematode metacercariae in the Republic of Korea. Korean J Parasitol..

[30] Thu ND, Dalsgaard A, Loan LTT, Murrell KD (2007). Survey for zoonotic liver and intestinal trematode metacercariae in cultured and wild fish in an Giang Province, Vietnam. Korean J Parasitol.

[31] van De N, Le TH, Murrell KD (2012). Prevalence and intensity of fish-borne zoonotic trematodes in cultured freshwater fish from rural and urban areas of northern Vietnam. J Parasitol.

[32] Petney TN, Andrews RH, Saijuntha W, Wenz-Mücke A, Sithithaworn P (2013). The zoonotic, fish-borne liver flukes *Clonorchis sinensis*, *Opisthorchis felineus* and *Opisthorchis viverrini*. Int J Parasitol.

[33] Yoon K-B, Lim H-C, Jeon DY, Park S, Cho S-H, Ju J-W (2018). Infection status with *Clonorchis sinensis* metacercariae in fish from Tamjin-gang (river) in Jeollanam-do, Republic of Korea. Korean J Parasitol.

[34] Chai J-Y, Sohn W-M, Na B-K, Yong T-S, Eom KS, Yoon C-H (2014). Zoonotic trematode metacercariae in fish from Phnom Penh and Pursat, Cambodia. Korean J Parasitol.

[35] Chuong NV, Tuan BV, Chau LV (1997). Several epidemiological characteristics of *Opisthorchis viverrini*. Malar Parasit Dis Prevent Bull.

[36] Charoensuk L, Ribas A, Chedtabud K, Prakobwong S (2022). Infection rate of *Opisthorchis viverrini* metacercariae in cyprinoid fish from the markets and its association to human opisthorchiasis in the local community in the Northeast Thailand. Acta Trop.

[37] Sukontason KL, Sukontason K, Boonsriwong N, Chaithong U, Piangjai S (2001). Intensity of trematode metacercariae in cyprinoid fish in Chiang Mal Province, northern Thailand. Southeast Asian J Trop Med Public Health.

[38] Traub RJ, Macaranas J, Mungthin M, Leelayoova S, Cribb T, Murrell KD, Thompson RCA (2009). A new PCR-based approach indicates the range of *Clonorchis sinensis* now extends to Central Thailand. PLOS Negl Trop Dis.

[39] Chontananarth T, Wongsawad C (2010). Prevalence of Haplorchis taichui in field-collected snails: a molecular approach. Korean J Parasitol.

[40] Chontananarth T, Tejangkura T, Wetchasart N, Chimburut C (2017). Morphological characteristics and phylogenetic trends of trematode cercariae in freshwater snails from Nakhon Nayok Province, Thailand. Korean J Parasitol.

[41] Kiatsopit N, Sithithaworn P, Kopolrat K, Namsanor J, Andrews RH, Petney TN (2016). Trematode diversity in the freshwater snail *Bithynia siamensis goniomphalos* sensu lato from Thailand and Lao PDR. J Helminthol.

[42] Sanil NK, Janardanan KP (2019). Four new species of virgulate xiphidiocercariae infecting the freshwater snail, *Bithynia* (Digoniostoma) *Pulchella* (Benson, 1836) in Malabar, Kerala. J Parasit Dis.

[43] Nath TC, Eom KS, Choe S, Islam S, Sabuj SS, Saha E (2022). Insights to helminth infections in food and companion animals in Bangladesh: occurrence and risk profiling. Parasite Epidemiol Control.

[44] Prakobwong S, Gunnula W, Chaipibool S, Nimala B, Sangthopo J, Sirivetthumrong N, Ribas A (2017). Epidemiology of in an endemic area of Thailand, an integrative approach. Helminthologia.

[45] Lin R-Q, Tang J-D, Zhou D-H, Song H-Q, Huang S-Y, Chen J-X (2011). Prevalence of *Clonorchis sinensis* infection in dogs and cats in subtropical southern China. Parasit Vectors.

[46] Dai RS, Li ZY, Li F, Liu DX, Liu W, Liu GH (2009). Severe infection of adult dogs with helminths in Hunan Province, China poses significant public health concerns. Vet Parasitol.

[47] Department of Livestock Services (DLS). Annual report on livestock. Farmgate, Dhaka, Bangladesh: Department of Livestock Services, Ministry of Fisheries and Livestock; 2023. https://dls.portal.gov.bd/sites/default/files/files/dls.portal.gov.bd/page/ee5f4621_fa3a_40ac_8bd9_898fb8ee4700/2023-07-23-12-04-afbcccb96f8b27d4bab6501aa8c2c2ff.pdf. Accessed 22 Feb 2024.

