# Supplementary figures and images for: Zoonotic human liver flukes, a type 1 biocarcinogen, in freshwater fishes: genetic analysis and confirmation of molluscan vectors and reservoir hosts in Bangladesh
Source: Infect Dis Poverty. 2024 Jun 1;13:40. doi: 10.1186/s40249-024-01209-0 (PMC11143560; doi:10.1186/s40249-024-01209-0)

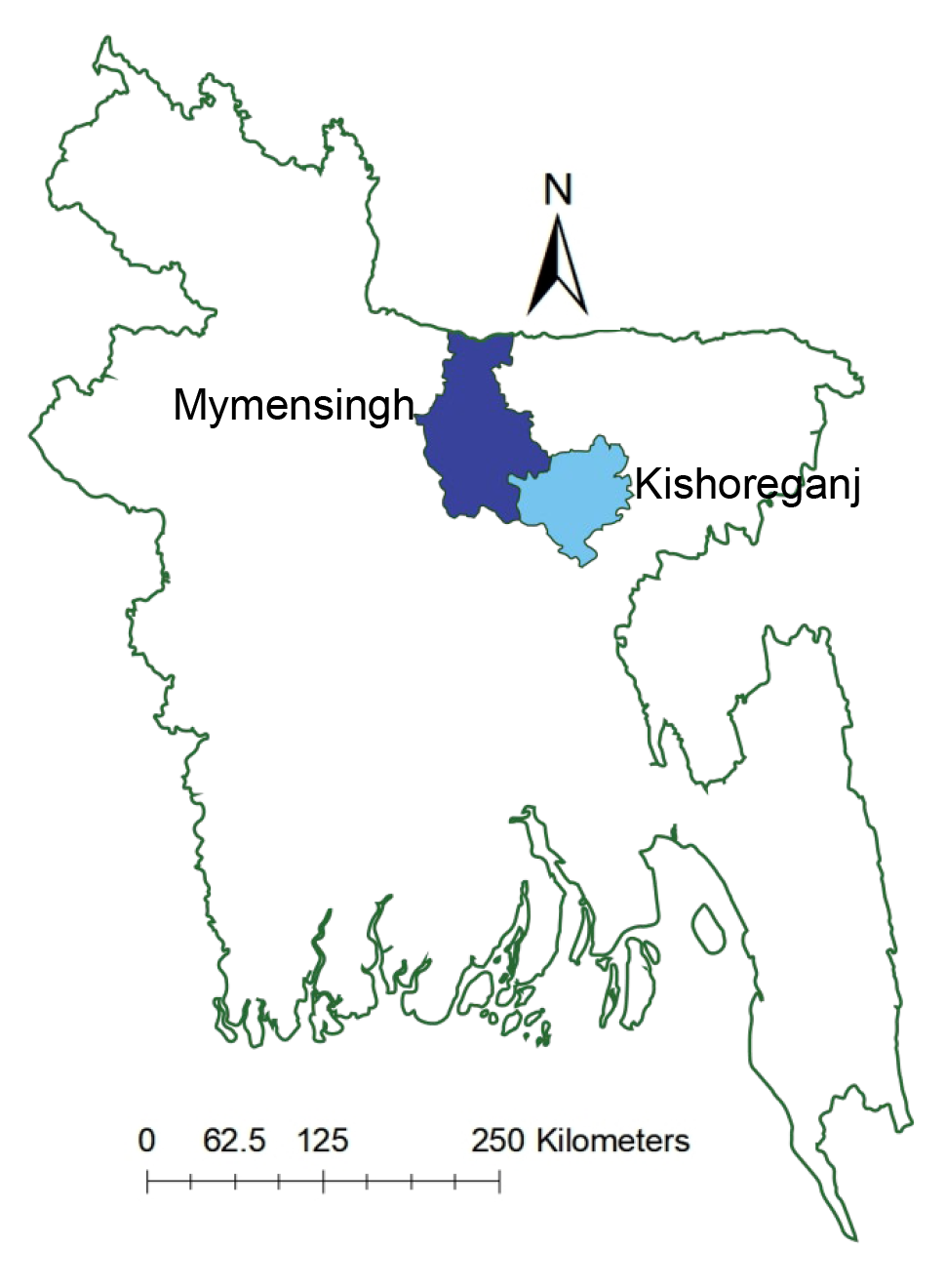

Supplement: Supplementary file 1 — Additional file 1: Supplementary Figure 1: A map showing study areas. [file 40249_2024_1209_MOESM1_ESM.png]

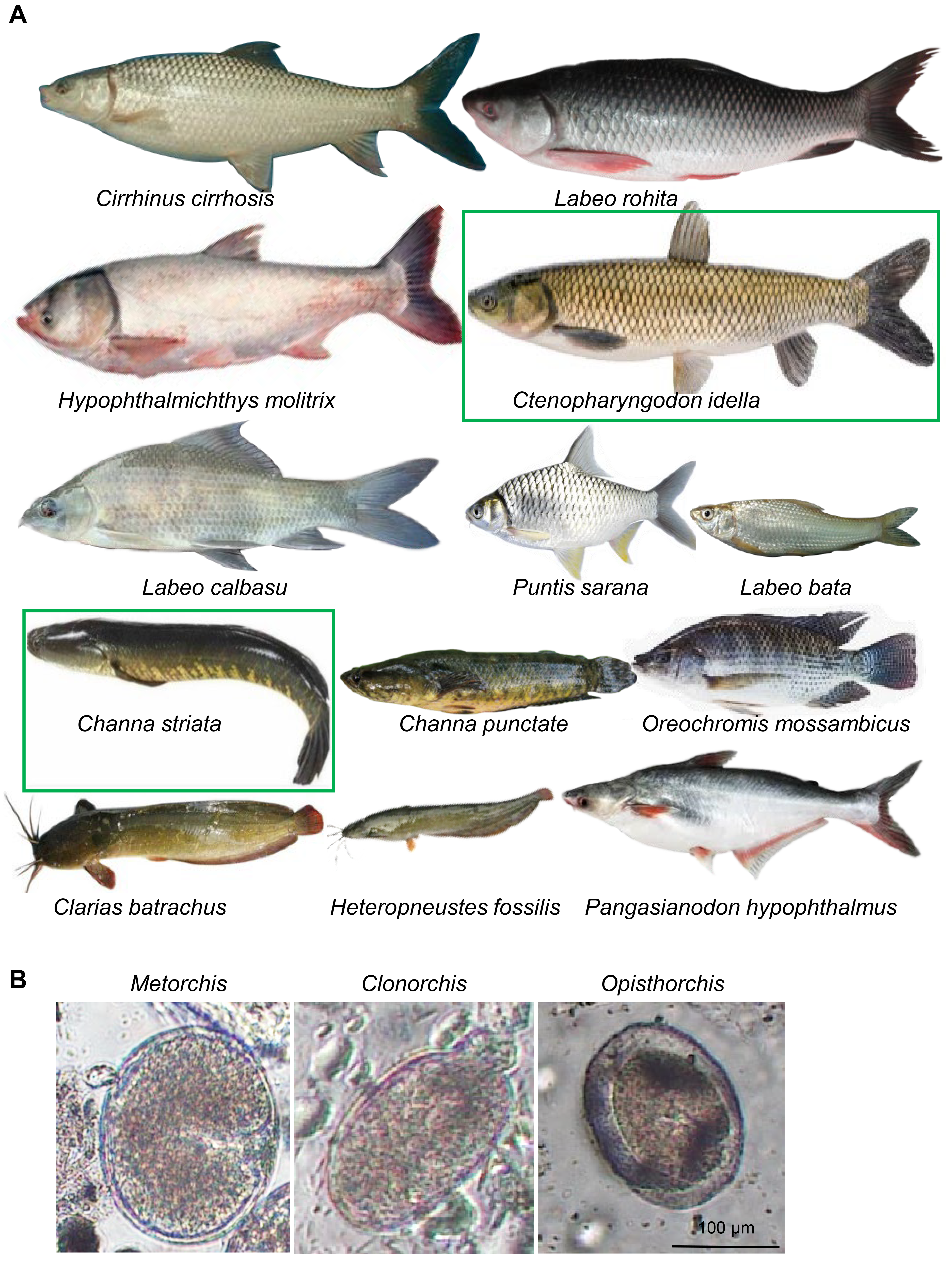

Supplement: Supplementary file 2 — Additional file 2: Supplementary Figure 2: Freshwater fishes studied and metacercariae recovered. (A) Large freshwater fishes infected with the metacercariae (MC) of human liver flukes (hLFs). Mrigal (Cirrhinus cirrhosus),rohu (Labeo rohita), silver carp (Hypophthalmichthys molitrix), grass carp (Ctenopharyngodon idella), orange fin labeo (L. calbasu), olive barb (Puntis sarana), bata (L. bata), striped snakehead (C. striata), spotted snakehead (Channa punctata), tilapia (Oreochromis mossambicus), walking catfish (Clarias batrachus), stinging catfish (Heteropneustes fossilis), and yellowtail catfish (Pangasianodon hypophthalmus) were collected and identified. Fishes within green boxes were free from infection. (B) Isolated MC of major hLFs. [file 40249_2024_1209_MOESM2_ESM.png]

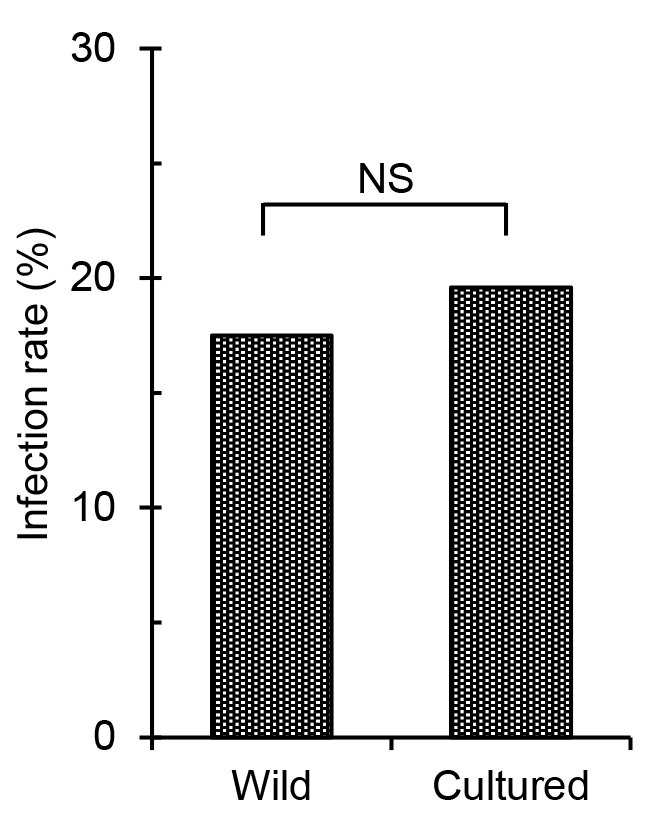

Supplement: Supplementary file 3 — Additional file 3: Supplementary Figure 3: Prevalence of metacercariae (MC) of human liver flukes (hLFs) in cultured and wild fishes. hLFs in cultured and wild fishes. [file 40249_2024_1209_MOESM3_ESM.png]
