# Supplementary material for: Zoonotic human liver flukes, a type 1 biocarcinogen, in freshwater fishes: genetic analysis and confirmation of molluscan vectors and reservoir hosts in Bangladesh
Source: Infect Dis Poverty. 2024 Jun 1;13:40. doi: 10.1186/s40249-024-01209-0 (PMC11143560; doi:10.1186/s40249-024-01209-0)
Supplement: Supplementary file 4 — Additional file 4. [file 40249_2024_1209_MOESM4_ESM.docx]

**Supplementary Table 1:** Size and weight of different species of fishes used

| **Family** | **Scientific name** | **Common name** | **Size (cm)** | **Weight (g)** |
| --- | --- | --- | --- | --- |
|  |  | **Bengali**  **(English)** | **Ranges**  **(Mean ± SD)** | **Ranges**  **(Mean ± SD)** |
| Cyprinidae | *Puntius ticto* | Punti  (Ticto barb) | 2.2-9.4  (6.6 ± 1.4) | 1.5-11.6  (4.6 ± 2.9) |
|  | *Esomus danricus* | Darkina  (Flying barb) | 1.5-5.6  (3.8 ± 0.6) | 0.2-1.5  (0.4 ±0.2) |
|  | *Colisa fasciata* | Kholisha  (Banded gourami) | 2.1-9.3  (7.9 ± 0.6) | 5.2-15.1  (9.7 ± 2.3) |
|  | *Cirrhinus reba* | Latchu  (Reba carp) | 2.4-14.3  (9.7 ± 4.2) | 3.2-20.9  (12.7 ± 5.9) |
|  | *Labeo rohita* (Native) | Rohu  (Rohu) | 18-44  (30.9 ± 6.2) | 254-1088  (455.7 ± 270.9) |
|  | *L. rohita (*Imported) | Rohu  (Rohu) | 40-60  (48.0 ± 6.5) | 1530-2900  (2529.1 ± 431.2) |
|  | *L. calbasu* | Kalibaus  (Orange fin labeo) | 12-39  (23.4 ± 7.3) | 90-1500  (388.6 ± 336.4) |
|  | *L. bata* | Bata  (Bata labeo) | 16-31  (22.4± 4.6) | 43-799  (202.6 ± 189.9) |
|  | *P. sarana* | Sarpunti  (Olive barb) | 10-33  (25.0 ± 7.4) | 233-616  (391.6 ± 143.3) |
|  | *Cirrhinus cirrhosis* | Mrigal  (Mrigal carp) | 26-50  (34.0±5.7) | 201-1433  (463.0 ± 310.9) |
|  | *Hypophthalmichthys molitrix* | Silver carp  (Silver carp) | 19-57  (35.4 ± 9.3) | 284-4000  (1107.6 ± 1200.0) |
|  | *Ctenopharyngodon idella* | Grass carp  (Grass carp) | 16-50  (36.7±10.6) | 201-2800  (1566.7 ± 1229.3) |
| Belonidae | *Xenentodon cancila* | Kakila  (Freshwater garfish) | 6.5-22  (17.5 ± 2.6) | 5.1-17.7  (10.5 ± 3.9) |
| Pangasiidae | *Pangasianodon hypophthalmus* | Pangas  (Yellowtail catfish) | 31-54  (40.6± 8.8) | 334-2000  (1405.4 ± 583.5) |
| Heteropneustidae | *Heteropneustes fossilis* | Singhi  (Stinging catfish) | 9-28  (18.9 ± 4.0) | 35-222  (77.6 ± 55.0) |
| Clariidae | *Clarias batrachus* | Magur  (Walking catfish) | 9-27  (16.8± 6.3) | 104-221  (134.2 ± 30.4) |
| Channidae | *Channa punctata* | Taki  (Spotted snakehead) | 6-22  (16.5 ± 4.0) | 58-389  (143.8 ± 99.9) |
|  | *C. striata* | Shol  (Stripped snakehead) | 12-30  (22.9± 6.4) | 201-700  (314.4 ± 130.6) |
| Cichlidae | *Oreochromis mossambicus* | Tilapia  (Tilapia) | 12-29  (19.9 ± 6.1) | 69-674  (244.3 ± 140.3) |
